# The screening score of Mini Nutritional Assessment (MNA) is a useful routine screening tool for malnutrition risk in patients on maintenance dialysis

**DOI:** 10.1371/journal.pone.0229722

**Published:** 2020-03-04

**Authors:** Els Holvoet, Karsten Vanden Wyngaert, Amaryllis H. Van Craenenbroeck, Wim Van Biesen, Sunny Eloot

**Affiliations:** 1 Renal Division, Department of Internal Medicine, Ghent University Hospital, Ghent, Belgium; 2 Department of Rehabilitation Sciences and Physiotherapy, Faculty of Medicine and Health Sciences, Ghent University, Ghent, Belgium; 3 Department of Nephrology, University Hospitals Leuven, Leuven, Belgium; 4 Laboratory of Experimental Medicine and Paediatrics, University of Antwerp, Antwerp, Belgium; Gazi University, Faculty of Health Sciences, TURKEY

## Abstract

**Purpose:**

Malnutrition is prevalent in patients on dialysis and is associated with morbidity and mortality. Nutritional status can be assessed by a variety of biochemical and physical parameters or nutritional assessment scores. Most of these methods are expensive or cumbersome to use and are not suitable for routine repetitive follow-up in dialysis patients. The Mini Nutritional Assessment (MNA) has a short form screening set (MNA-SF), which would be suitable as a screening tool, but has not been validated yet in dialysis patients. We aimed to assess whether the MNA is an appropriate tool for identifying nutritional problems in dialysis patients.

**Method:**

MNA, routine biochemistry, physical parameters, comorbidities were assessed in cross-sectional multicentric cohorts of hemodialysis and peritoneal dialysis patients with a longitudinal follow up of 2 years for mortality.

**Results:**

In this cohort of 216 patients, mortality was 27.3% at a follow up of 750±350 days. The mean MNA-SF score was 9.9±1.8, with 30.1%, 59.3% and 10.6% of patients categorized as having normal nutritional status, at risk for malnutrition and malnourished, respectively. The screening score was associated with mortality (HR 0.86, 95% CI 0.75–0.98 per point). With normal nutrition as reference, adjusted mortality was 2.50 (95% CI 1.16–5.37) and 3.89 (95% CI 1.48–10.13) for patients at risk for malnutrition and with malnutrition, respectively. After recalibrating the MNA full score for the specificity of some of its domains for dialysis patients, the MNA-SF had a good sensitivity and specificity for not being well nourished (0.95 and 0.63 respectively) in the full score, and a high negative predictive value (0.91).

**Conclusion:**

The MNA-SF is independently associated with 2 year mortality in dialysis patients. It has a high negative predictive value for excluding being at risk or having malnutrition in the full score. Therefore, it can be advocated as a screening tool for nutritional status in dialysis patients.

## Introduction

Malnutrition is an important and prevalent problem affecting 16% to 70% of dialysis patients, depending on the study population and assessment procedure [1–3]. In dialysis patients, insufficient nutritional intake, compromised clinical well-being, comorbidity and dialysis related factors can lead to poor nutritional status [4–6]. Poor nutritional status is on its turn associated with impaired functional capacity, increased risk of complications, lower quality of life and limited survival [3, 7–9]. Therefore it seems important to identify patients at risk for malnutrition already at an early stage, in order to start interventions to improve their nutritional status and clinical and mental outcome. Most guidelines therefore recommend to evaluate nutritional status in dialysis patients [10–12]. Nutritional status of dialysis patients can change rapidly, so frequent evaluation is needed. Objective standardized assessment of nutritional status in dialysis patients might be laborious, logistically difficult, time consuming and thus expensive, so there is a need for an accurate and reliable screening tool that can routinely be applied at the bedside on a repetitive basis. An adequate nutritional assessment tool should meet several essential criteria: (1) diagnose malnutrition accurately, (2) detect changes in nutritional status over time, (3) be associated with morbidity and mortality and (4) evaluate the impact of a nutritional intervention [9]. Several scoring systems have been developed to estimate nutritional status based on readily available parameters. The Mini Nutritional Assessment score (MNA) is probably the most widely used and best validated score in different elderly populations and settings [13–15]. The tool consists of a 6-item short form screening tool (MNA-SF) included in the 18-item long form scale (MNA-LF). According to the instructions of the MNA score, no further evaluation by the full score is needed when patients are rated as “normal nutrition” by the MNA-SF, as the score has a high negative predictive value for malnutrition. This makes the tool ideally suited for rapid screening, as would be the case for regular follow-up in dialysis patients. However, when the MNA-SF indicates ‘at risk of malnutrition’ or ‘malnourished’, further assessment by the full version is needed to confirm the diagnosis. Research shows the MNA has strong sensitivity, specificity and predictive value for malnutrition [13, 14, 16]. Several of the items in the MNA scale are however likely to have a different meaning in a dialysis cohort as compared to a more general population, such as the items on protein intake, fluid intake or consumption of fruit and vegetables. As a consequence, validation and eventual recalibration of the MNA score in a dialysis cohort is necessary.

The aim of this study is therefore 1/ to evaluate the association of the MNA-SF and MNA-LF with 2 year mortality in the dialysis population; 2/ to evaluate the negative predictive value for risk for malnutrition/malnutrition of the score “normal nutritional status” of the MNA-SF; 3/ to explore eventual need for recalibration of the MNA-LF.

## Methods

### Study design and subjects

For this cross-sectional multicentric study with longitudinal follow up, all consecutive adult (>18 years) dialysis patients of the hemodialysis units of the Ghent University Hospital and Antwerp University Hospital (Belgium) were eligible. The Ghent University Hospital has 3 large satellite hemodialysis units, a unit for nocturnal hemodialysis and a peritoneal dialysis programme, and also these patients were eligible for inclusion. Assessment of patients with acute inflammatory disease and/or who underwent recent (<3 weeks) surgery was postponed until their condition was stabilized. Pregnancy, substantial cognitive impairment and not being able to understand Dutch were exclusion criteria. Patients were included in different waves, starting from November 2015, and followed up prospectively until death or end of study (January 9, 2019). The study was approved by the Ethical Committee of the Ghent University Hospital and the Ethical Committee of the Antwerp University Hospital and written informed consent was obtained from each participant (project number Ghent B670201525559, and Antwerp B300201422642). Registration number on clinicaltrial.gov: NTC03910426.

### Questionnaires and scores

#### The Mini Nutritional Assessment (MNA)

The MNA is a validated instrument initially developed to assess nutritional status in elderly patients and is mainly indicated for research settings [13, 17]. The tool contains 18 items and evaluates 4 different aspects: anthropometric assessment (body mass index (BMI), weight loss, and arm and calf circumferences); general assessment (lifestyle, medication, mobility and presence of signs of depression or dementia); short dietary assessment (number of meals, food and fluid intake and autonomy of feeding); and subjective assessment (self-perception of health and nutrition). By adding up the scores, labelled as *MNA-LF*, individuals can be divided in 3 groups using threshold values of <17 for ‘malnourished’, 17–23.5 for ‘at risk of malnutrition’ and ≥ 24 for ‘normal nutritional status’, with a maximum total score of 30 points [13]. As we made the hypothesis that a recalibration of the MNA-LF in patients on dialysis might be warranted, we used also different cut offs for categorization. First, a categorization whereby the median score of patients not in the ‘normal nutritional status’ group was used to split them further into a ‘low risk of malnutrition’ and a ‘high risk of malnutrition’ group. This score was labelled as *MNA-LF-new*. Second, taking into account that in dialysis patients the items “intake of fruit and vegetables” and “amount of fluid intake” could be governed by factors other than nutritional intake, we lowered the cut off for “normal nutritional status” to 22 (instead of 24). This score was labelled as *MNA-LF-ESKD (MNA-LF-end stage kidney disease)*. The first six items (together 14 points) of the MNA can be used as a short screening tool to classify patients as having a normal nutritional status, a risk for malnutrition and malnutrition, with threshold values of >11, 8 and 8>, respectively. This score was labelled as *MNA-SF*. According to the instructions of the screening test, no further evaluation is needed when patients classify as “normal nutritional status”. For the purpose of this study, we have however also performed the MNA-LF in patients with normal nutritional status in the short form, in order to be able to assess re-categorization between MNA-SF and the three MNA-LF scores.

#### Davies stoke score

Comorbidity was assessed using the Davies Stoke Score [18]. This score assigns 1 point for each of the following conditions: malignancy, ischemic heart disease, peripheral vascular disease, left ventricular dysfunction, diabetes mellitus, systemic collagen vascular disease, and other significant pathologies that have an impact on survival in the general population. The grade of comorbidity is derived directly from the total score; Grade 0 (low risk) is a zero score, grade 1 (medium risk) is a score of 1–2, and grade 2 (high risk) a cumulative score of ≥ 3. All parameters of the scores were surveyed by a trained research nurse and entered directly in a dedicated database. Diabetes was defined as patients taking one or more glycaemia lowering drugs or diet.

#### Biomarkers

Biochemical data were retrieved from the electronic medical records on a date as close as possible to the data of inclusion, with a maximum difference of 4 weeks. Only data collected at the midweek pre-dialysis session were included.

#### Outcome

The survival duration data were calculated from the time of inclusion in the study cohort to death (event) or the end of the observation period, January 9, 2019 (censored). Final status of all patients was derived from electronic medical records as no patients were lost to follow up during the observation period.

#### Methodological quality

Methodological quality was assessed using the Newcastle-Ottowa Quality Assessment Form for cohort studies (NOS) [19].

### Statistical analysis

Continuous variables were summarized as mean ± standard deviation (SD). Chi square or One way ANOVA was used to compare variables over subgroups.

Survival in different groups were calculated as time to event using Cox proportional hazard models, and as two year survival rate using logistic regression. Results are presented as hazard ratios (HR) with 95% confidence intervals (CI).

## Results

Overall, 216 patients (63.9% men, 36.2% women, 38.9% with diabetes, age 67±15 years) were included. The sample is representative for the dialysis population in Flanders [20], with 90.3% of them living independently, 94.9% treated with hemodialysis and 5.1% with peritoneal dialysis.

**Fig 1:**
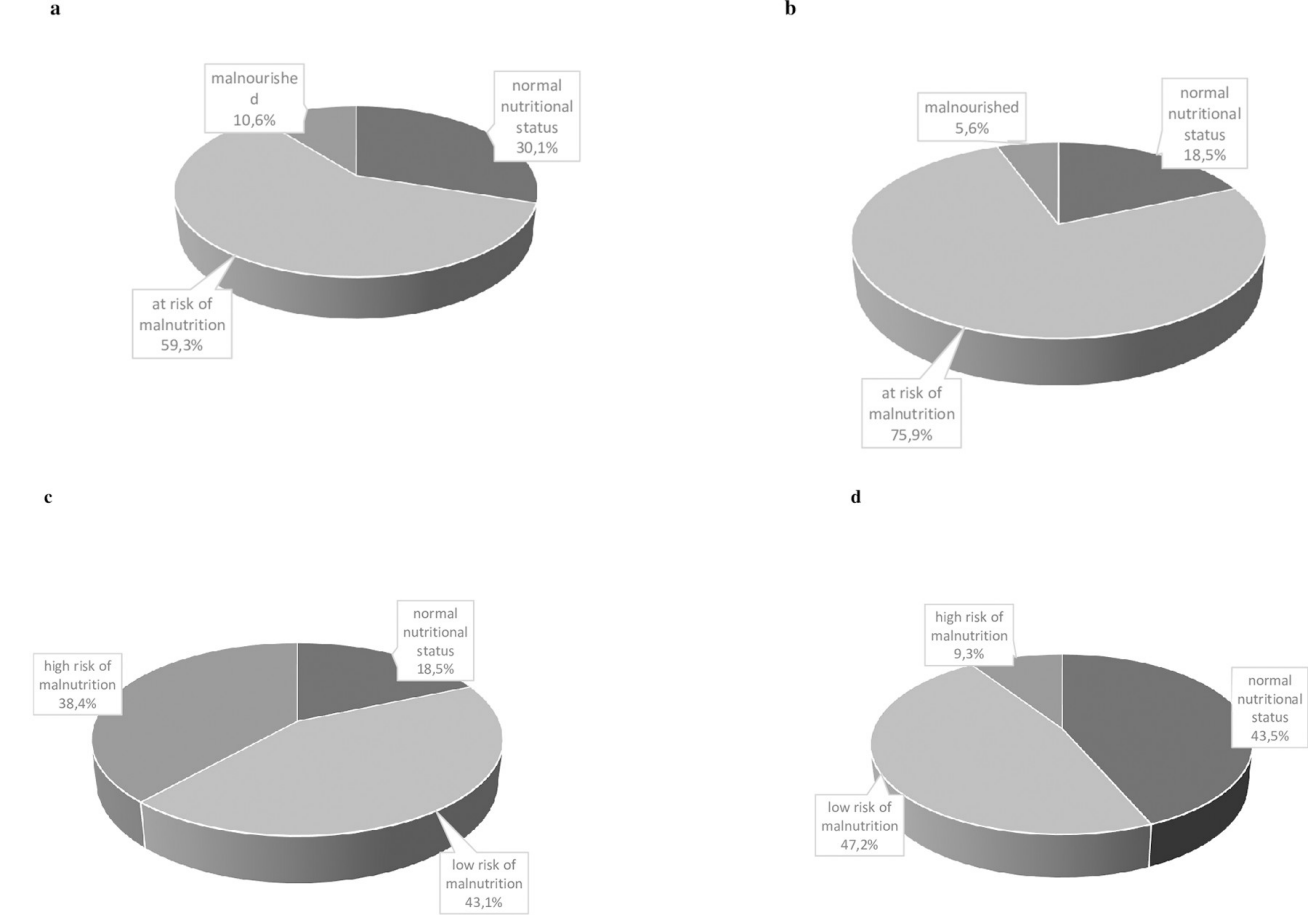
**(A)** Categorization according to the standardized MNA-SF score. **(B)** Categorization according to the standardized MNA-LF score. **(C)** Categorization according to the MNA-LF-new score. **(D)** Categorization according to the MNA-LF-ESKD score.

The mean MNA-SF score was 9.9±1.8, with 30.1%, 59.3% and 10.6% of patients being categorized as having normal nutritional status, at risk for malnutrition and malnourished, respectively **(Fig 1A)**.

The mean MNA-LF score was 21.0±2.7, with 18.5%, 75.9% and 5.6% of patients being categorized as having a normal nutritional status, at risk for malnutrition and malnourished, respectively **(Fig 1B)**.

**Fig 2:**
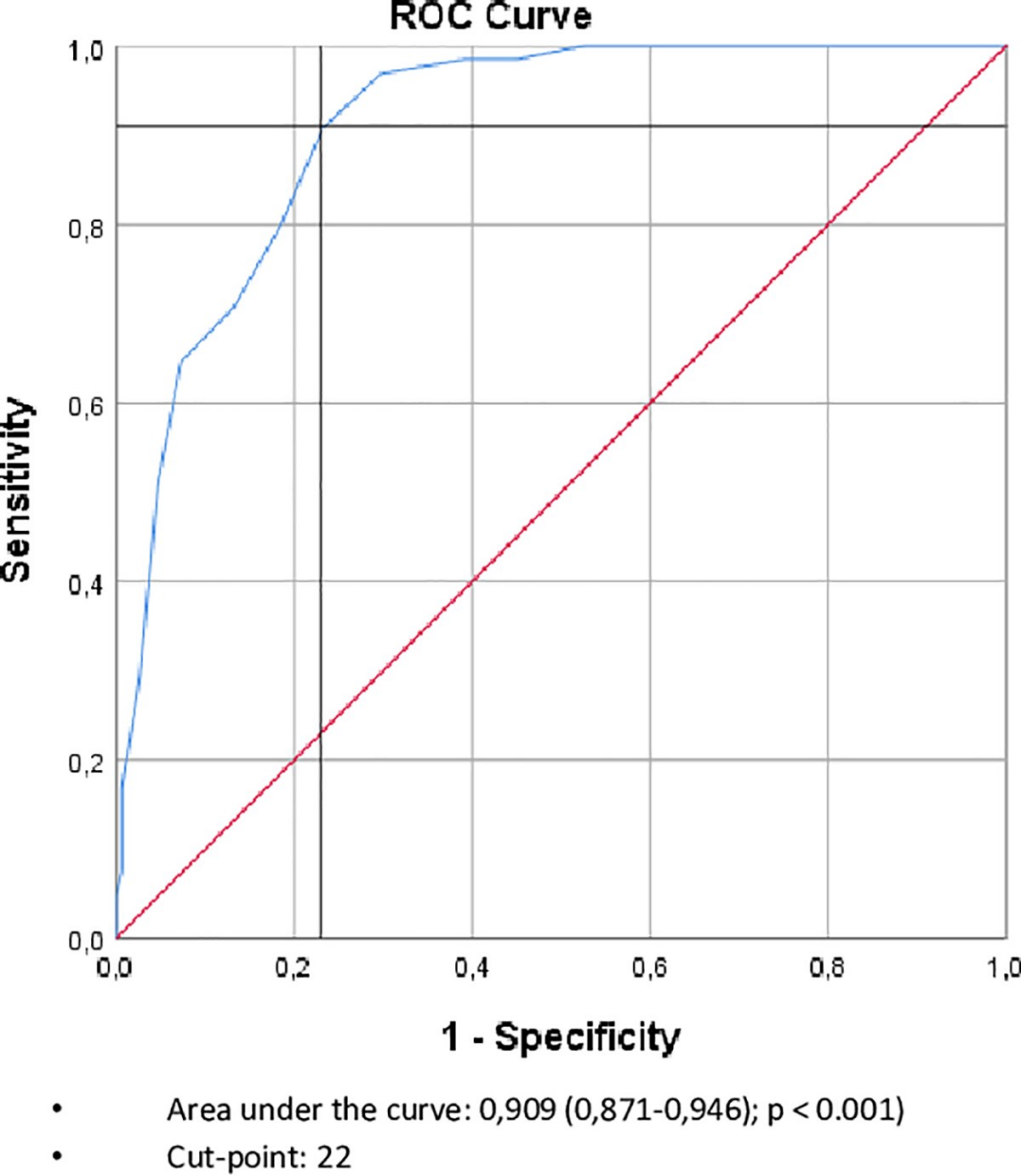
ROC curve for the MNA-LF using dichotomized MNA-SF (normal nutritional status versus at risk/malnourished) as referent.

When applying the alternative categorizations, 18.5%, 43.1% and 38.4% of the patients had a normal nutritional status, a low risk and a high risk of malnutrition, respectively, with the MNA-LF-new categorization **(Fig 1C)**, and 43.5%, 47.2% and 9.3% had a normal nutritional status, a low risk and a high risk of malnutrition, respectively, with the MNA-LF-ESKD categorization **(Fig 1D)**. Discrimination between normal nutritional and non-normal nutritional status, defined as at risk for or being malnourished, based on the Receiving Operating Characteristic Curve (ROC) resulted in an area under the curve of 0.909 (0.871–0.946) (p< 0.001), with a best fit point of 22 **(Fig 2)** which corresponds to our pre-defined re-categorization proposal MNA-LF-ESKD.

After this recalibration, the MNA-SF had a good sensitivity, and specificity for not being well nourished (so either at risk for malnutrition or malnourished) (0.95 and 0.63, respectively), and had a high negative predictive value of 0.91.

**Fig 3:**
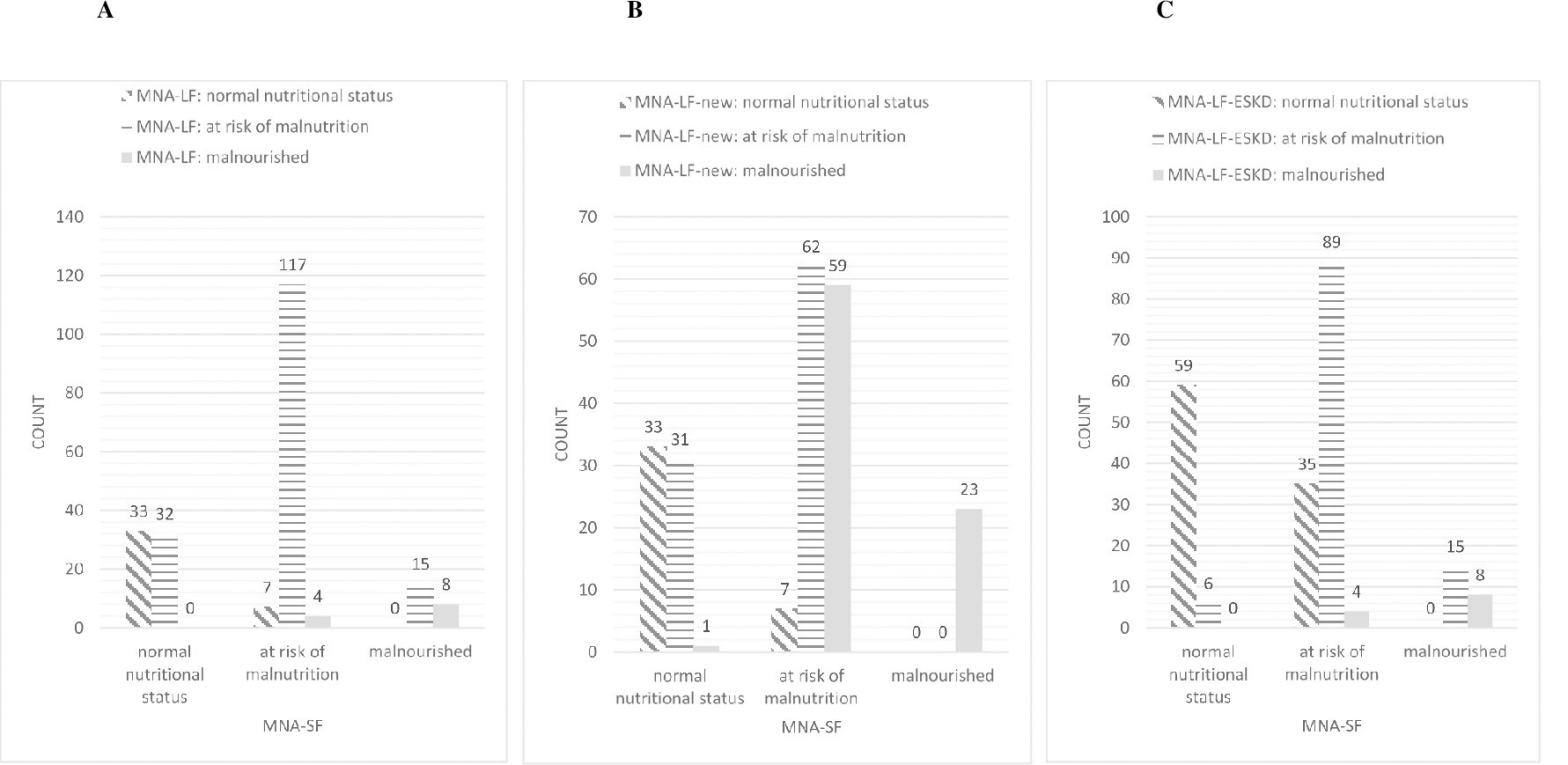
**(A)** Re-categorization between MNA-SF and MNA-LF. **(B)** Re-categorization between MNA-SF and MNA-LF-new. **(C)** Re-categorization between MNA-SF and MNA-LF-ESKD.

Re-categorization between MNA-SF and MNA-LF, MNA-LF-new and MNA-LF-ESKD is presented in **Fig 3A, 3B and 3C**, respectively.

**Table 1** shows the characteristics of the study population and this separated for the different malnutrition subgroups according to MNA-SF.

**Table 1:** Characteristics of the study population in the different malnutrition subgroups based on MNA-SF.

characteristics	Total (N = 216)	Normal nutrition (N = 65)	At risk of malnutrition (N = 128)	malnourished (N = 23)	P-value
	Frequency (%)	Frequency (%)	Frequency (%)	Frequency (%)	
**Age**					
<**65**	75 (34.7)	25 (38.6%)	42(32.8%)	8 (34.8%)	0.569
**65–74**	57 (26.4)	17 (26.2%)	33 (25.8%)	7 (30.4%)	
**75–85**	69 (31.9)	22 (33.8%)	41 (32.0%)	26 (26.1%)	
**>85**	15 (6.9)	1 (1.5%)	12 (9.4%)	2 (8.7%)	
**Mean age**	67.2±15.7	65.9±1.3	67.8±16.8	67.1±16.2	0.720
**Gender**	2				
**Male**	138 (63.9)	46 (70.8%)	76 (59.4%)	16 (69.6%)	0.248
**Female**	78 (36.1%)	19 (29.2%)	52 (40.6%)	7 (30.4%)	
**Renal replacement therapy**					
**Hemodialysis**	205 (94.9%)	61 (93.8%)	122 (95.3%)	22 (95.7%)	0.895
**Peritoneal dialysis**	11 (5.1%)	4 (6.2%)	6 (4.7%)	1 (4.3%)	
**Davies Stoke Comorbidity score**					
**Low risk**	36 (16.7)	91 (13.8%)	24 (18.8%)	3 (13.0%)	0.169
**Medium risk**	103 (47.7)	38 (58.5%)	57 (44.5%)	8 (34.8%)	
**High risk**	77 (35.6)	18 (27.7%)	47 (36.7%)	12 (52.2%)	
**Diabetic status**					
**Nondiabetic**	132 (61.1%)	41 (63.1%)	78 (60.9%)	13 (56.5%)	0.856
**Diabetic**	84 (38.9)	24 (36.9%)	50 (39.1%)	10 (43.5%)	
**BMI**					
**<18.5**	5 (2.3)	0 (0%)	4 (3.1%)	1 (4.3%)	0.020
**18.5–24.9**	93 (43.1)	18 (27.7%)	64 (50.0%)	11 (47.8%)	
**25–29.9**	69 (31.9)	29 (44.6%)	36 (28.1%)	4 (17.4%)	
**>30**	49 (22.7)	18 (27.7%)	24 (18.8%)	7 (30.4%)	
**Living independently**					
**Yes**	195 (90.3%)	61 (93.8%)	115 (89.8%)	19 (82.6%)	0.285
**No**	21 (9.7%)	4 (6.2%)	13 (10.2%)	4 (17.4%)	
**C-reactive protein (mg/L) (Mean, SD)**		9.6±16.0	9.3±16.4	21.1±52.9	0.595
**Serum total protein (g/L) (Mean, SD)**		65.7±5.5	65.0±6.5	65.6±6.6	0.738

The majority of the study population took more than 3 different medications (95.4%). According to BMI thresholds proposed by the WHO, only 43.1% of patients had a normal body mass index, whereas 2.3% of the patients was underweight, 31.9% was overweight, and 22.7% had obesity. According to the Davies Stoke Comorbidity scale, 16.7% of the study population had a low mortality risk, 47.7% a medium risk and 35.6% a high risk.

During a mean follow up of 750±350days, 59 (27.3%) patients died. Patients who died were older (74.4±12.7 versus 64.3±15.8 years in the survivors, p<0.001), had a lower BMI (25.4±4.1 versus 26.9±5.7kg/m^2^, respectively) and a worse Davies Stoke Comorbidity score (p = 0.007) **(Table 2)**.

**Table 2:** Characteristics of the study population in different subgroups based on the mortality status.

characteristics	Alive (N: 59; 27.3%)	Death (N: 157; 72.7%)	P-value
	Mean±SD	Mean±SD	
**Age**	64.3±15.8	74.4±12.7	<0.001
**BMI**	26.9±5.7	25.4±4.4	0.042
**Davies stoke score**	1.8±1.4	2.4±1.3	0.007

Outcomes of univariate Cox proportional hazard regression for relevant parameters are presented in **Table 3**.

**Table 3:** Univariate Cox regressions for mortality.

Variable	Exp Beta	95% CI
**Age (years)**	1.05	1.03–1.08
**Gender (female)**	0.94	0.55–1.61
**MNA-LF score (point)**	0.88	0.80–0.97
**MNA-LF category**	1.66	1.05–2.62
**MNA-LF-ESKD category**	1.67	1.15–2.44
**MNA-LF-new category**	1.56	1.07–2.28
**MNA-SF score (point)**	0.86	0.75–0.98
**MNA-SF category**	1.92	1.26–2.91
**MNA-SF normal vs other**	3.03	1.44–6.40
**Davies score (point)**	1.25	1.06–1.48
**Diabetes (yes vs no)**	1.35	0.81–2.25
**Living independently (yes vs no)**	0.41	0.20–0.84
**Consumption of fruit and vegetables**	0.90	0.62–1.30
**Fluid intake**	0.70	0.39–1.25
**Mid arm circumference category**	0.77	0.52–1.14
**Calve circumference category**	0.41	0.24–0.70
**Serum protein (g/l)**	0.98	0.94–1.03
**C-reactive protein (mg/l)**	1.00	0.99–1.01

In a Cox regression model with age, gender, diabetes and Davies Stoke Score forced in the model, nutritional status as assessed by the MNA-SF categorization was better associated with mortality than MNA-LF, MNA-LF-new MNA-LF-ESKD or MNA-SF as continuous variable, and this irrespective of whether the model was run as forward or backward entry mode **(final model in Table 4)**.

**Table 4:** Multivariate Cox regression.

Variable	Exp Beta	95% CI
**Gender (female vs male)**	1.05	0.60–1.83
**Age (years)**	1.05	1.02–1.08
**Diabetes (no vs yes)**	0.95	0.52–1.75
**MNA-SF**		
**Normal nutritionals status (reference)**	1	
**At risk of malnutrition**	2.50	1.16–5.37
**Malnourished**	3.89	1.48–10.13
**Davies Stoke Score**	1.05	0.85–1.32

In the initial starting model also Davies Stokes Score, MNA-LF score, MNA-LF-ESKD and MNA-LF-new were added, but not retained in forward or backward modeling.

**Fig 4:**
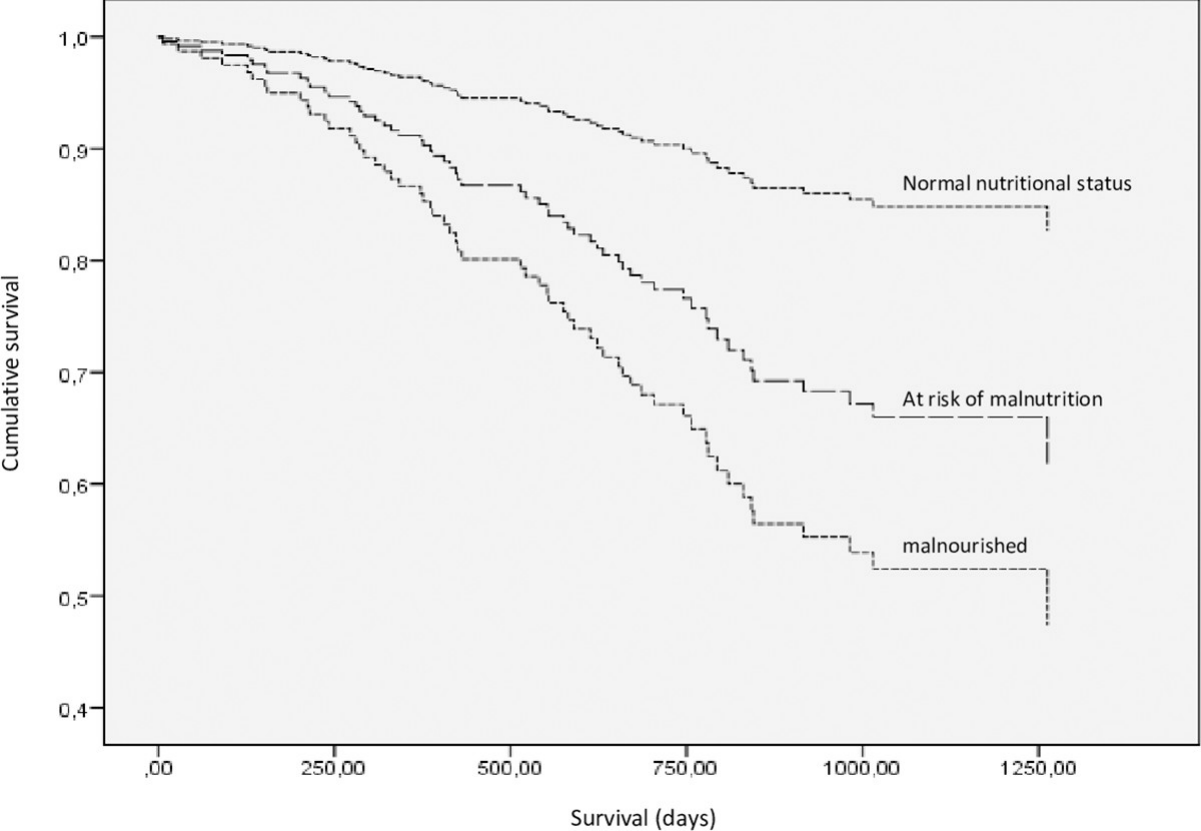
Survival in function of MNA-SF score, adjusted for age, gender and Davies Stoke Score.

**Fig 4** represents survival over time adjusted for gender, age, diabetes status and Davies Stoke Score according to MNA-SF categorization.

In a binary logistic regression model for mortality at 2 years, increasing MNA-SF scores were associated with increasing risk of death, even after adjusting for age, gender, diabetes and Davies Stoke Score **(Table 5)**.

**Table 5:** Multivariate binary logistic regression for mortality at 2 years.

Variable	Exp Beta	95% CI
**Gender (female vs male)**	1.31	0.61–2.82
**Age (years)**	1.06	1.02–1.09
**MNA-SF**		
**Normal nutritional status (reference)**	1	
**At risk of malnutrition**	3.86	1.39–10.70
**Malnourished**	5.00	1.43–18.78
**Diabetes**	0.83	0.36–1.91
**Davies Stoke Score**	1.15	0.84–1.56
**Constant**	0.004	

## Discussion

Our results indicate that MNA is strongly associated with mortality in patients on dialysis, and can thus be seen as a valid assessor of nutritional problems in this population. Categorization based on the MNA-SF score and on the MNA-LF score correspond quite well, but only when the specific features of the dialysis population are taken into account by slightly recalibrating the cut-offs for the MNA-LF score as was also confirmed by ROC curve analysis. The MNA-SF score can be performed easily and rapidly at bedside, and is thus an ideal screening tool to be used on a regular basis in the dialysis patient to detect patients at risk for malnutrition.

Malnutrition is a common problem in patients on dialysis, and is strongly associated with mortality. Most scoring systems for malnutrition use parameters that might be less reliable or less representative in patients on dialysis, and their relation with mortality in this population might be counterintuitive. The most striking example is the obesity paradox that higher BMI appears to be protective in patients on dialysis [5]. Therefore, it is essential that malnutrition scores are validated in dialysis patients, using mortality as outcome. Few studies aimed to validate the MNA in ESKD population [21–24], and only 2 studies carried out survival analysis. Brzozko et al. [23] identified a higher hazard of death in PD patients categorized by the MNA-LF as at risk of malnutrition or malnourished (HR 5.7, 95% CL 4.1–7.2). These results were confirmed after adjustment for demographics, comorbidities and laboratory values. However, the authors only assessed the nutritional status on a sample of 41 patients. Santin et al. [24] examined the predictive validity of the MNA-SF in an elderly HD population (≥ 60 years) and reported a higher mortality risk in patients with a risk of malnutrition and malnourishment in the crude and adjusted models (HR 5.53, 95% CL 1.34–4.77: adjusted for age, gender, dialysis vintage and diabetes). Our results indicate that MNA performs well, and is associated with survival in a dose-dependent way. Previous work has already validated Subjective Global Assessment (SGA) [25] as a tool to assess nutritional status in other settings, and also in the dialysis population [26]. In most studies, SGA was associated with mortality [24, 27, 28]. For these reasons, SGA is recommended by European Renal Best Practice as a good tool to screen nutritional status in the elderly [10]. However, SGA still can be criticized for its subjectivity, and thus potential problems for reproducibility [28, 29], and several authors report different results on the association of SGA with more objective nutritional markers [30, 31]. Further SGA takes some time and expertise to be evaluated, making it less suitable in fact for routine use as a regular screening tool in a busy dialysis unit. For use in patients on dialysis, another Protein Energy Wasting (PEW) score was developed based on the ARNOS cohort [32]. This score, graded from 0 (worse) to 4 (best) was derived from 4 different aspects of nutrition: serum albumin, body mass index, a normalized serum creatinine value, and protein intake as assessed by normalized Protein derived Nitrogen Appearance (nPNA) [10, 11]. This score was associated with survival at 3.5 year, and this in a dose dependent manner: there was a reduction in survival (5%-7%; P < 0.01) for each unit decrement in the score grade. In addition, the 6-month variation of this PEW score on reassessment also strongly predicted patients' survival (P<0.01) [32]. In theory, repetitive determination of this PEW score may be of help to better identify subgroups of patients at risk for malnutrition, and in which nutrition support should be enforced. However, assessment of nPNA is in practice rather laborious, and it is therefore unclear whether this score can be used in everyday practice.

As nutritional status of dialysis patients can change rapidly over time, regular screening and follow up is essential. However, most nutritional assessment tools are cumbersome, need specialized personnel or equipment, or are simply time consuming, and can therefore not be used as repetitive, routine tools.

Our results indicate that MNA performs as well when only the short form is performed. This short screening part consists of 6 questions that can easily and rapidly be completed at bedside, without any additional tool or equipment. Further refinement in patients at risk could be done by using e.g. a PEW scoring as based on ARNOS cohort [32].

Categorization of nutritional status as based on the MNA short form appeared to have the strongest correlation with mortality, even stronger than the full score of MNA. This can be explained by the specific situation of dialysis patients, resulting that some domains in the long form of the MNA score do not add to its discriminative power. The items “fluid intake” and “consumption of fruit and vegetables” are not representative in dialysis patients, as these are not related to decrease in food intake, but are forced by dietary restrictions linked to being a dialysis patient. The item “takes more than 3 drugs/day” is not discriminatory in dialysis patients, as this is the case in nearly all patients. This assumption is supported by studies finding low agreement between SGA and MNA [21, 22, 24]. The observed low Cohen’s kappa scores could be attributed to the generalizability of the two scores outside their original target population. The SGA has already been validated extensively in adults on hemodialysis and therefore is recognized as being applicable in this population. The MNA however was developed for the elderly in general and is thus not on itself adapted to an ESKD population. Therefore, the categorization based on the MNA-LF score probably needs some recalibration in the setting of dialysis patients. Based on Receiver Operating Characteristics analysis and on the reasoning that it is in fact for most dialysis patients impossible to get 2 points (fluid, and fruit and vegetables), it seems reasonable to use 22 points as the cut-off between normal nutritional status and being at risk for malnutrition. When this categorization criterion is applied, the screening score and full score appear to be very well matched, with very few patients re-categorized. This further strengthens that the screening score is very performant to identify patients with a questionable nutritional status, and that a normal score is reassuring.

The strengths of this study are the availability of the hard outcome of mortality, the prospective collection of data, and the relatively long follow up time, with a mean of two years. Our population is also representative for the average dialysis unit in Flanders, with a good mix of patients on daytime dialysis in hospital as well as in satellite dialysis units, patients on nocturnal dialysis and peritoneal dialysis. Further, with a NOS-score of 8 point, our study can be considered as a ‘good quality’ study when applying the criteria suggested by McPheeters et al. [33] (**S1 Material)**.

A limitation is of course the observational nature of our study. It is accordingly not possible to assess whether improving nutritional status in patients at risk for malnutrition will also effectively result in improvement of outcomes.

In conclusion, maintaining a good nutritional health in patients on dialysis could be a crucial element in the prevention of deteriorating well-being, and therefore nutritional status requires close attention. By applying survival analysis, this current study indicates that MNA-SF is an appropriate assessment tool for identifying nutritional problems in dialysis patients. Moreover the feasibility of the instrument makes it easy to integrate nutritional assessment in routine care.

## Supporting information

S1 Checklist STROBE statement—Checklist of items that should be included in reports of observational studies. (DOCX)

S1 Dataset (XLSX)

S1 Meterial Newcastle—Ottawa quality assessment scale cohort studies. (DOCX)
