# Supplementary material for: The screening score of Mini Nutritional Assessment (MNA) is a useful routine screening tool for malnutrition risk in patients on maintenance dialysis
Source: PLoS One. 2020 Mar 4;15(3):e0229722. doi: 10.1371/journal.pone.0229722 (PMC7055863; doi:10.1371/journal.pone.0229722)
Supplement: S1 Meterial — (DOCX) [file pone.0229722.s003.docx]

**Supplementary material 1**

**NEWCASTLE - OTTAWA QUALITY ASSESSMENT SCALE**

**COHORT STUDIES**

Note: A study can be awarded a maximum of one star for each numbered item within the Selection and Outcome categories. A maximum of two stars can be given for Comparability

- **Selection**

1) Representativeness of the exposed cohort: *

**a) truly representative of the average dialysis population in the community ***

b) somewhat representative of the average ______________ in the community *****

c) selected group of users eg nurses, volunteers

d) no description of the derivation of the cohort

2) Selection of the non exposed cohort *

a) **drawn from the same community as the exposed cohort ***

b) drawn from a different source

c) no description of the derivation of the non exposed cohort

3) Ascertainment of exposure *

a**) secure record (eg surgical records) ***

b) **structured interview** *****

c) written self report

d) no description

4) Demonstration that outcome of interest was not present at start of study *

a) **yes ***

b) no

- **Comparability**

1) Comparability of cohorts on the basis of the design or analysis *

a) **study controls for age, gender, Davies comorbidity score, diabetes ***

b) study controls for any additional factor ***** (This criteria could be modified to

indicate specific control for a second important factor.)

- **Outcome**

1) Assessment of outcome*

a) independent blind assessment *****

b) **record linkage ***

c) **self report**

d) **no description**

2) Was follow-up long enough for outcomes to occur *

a) **yes** **(750 ±350 days) ***

b) no

3) Adequacy of follow up of cohorts*

a) **complete follow up - all subjects accounted for ***

b) subjects lost to follow up unlikely to introduce bias - small number lost - > __ %

(select an adequate %) follow up, or description provided of those lost) *****

c) follow up rate < ____% (select an adequate %) and no description of those lost

d) no statement

**Note**: Selected items are in bold
